# Using arborescences to estimate hierarchicalness in directed complex networks

**DOI:** 10.1371/journal.pone.0190825

**Published:** 2018-01-30

**Authors:** Michele Coscia

**Affiliations:** 1 Center for International Development, Harvard University, Cambridge, MA, United States of America; 2 Naxys Department of Mathematics, University of Namur, Namur, Belgium; Georgia Institute of Technology, UNITED STATES

## Abstract

Complex networks are a useful tool for the understanding of complex systems. One of the emerging properties of such systems is their tendency to form hierarchies: networks can be organized in levels, with nodes in each level exerting control on the ones beneath them. In this paper, we focus on the problem of estimating how hierarchical a directed network is. We propose a structural argument: a network has a strong top-down organization if we need to delete only few edges to reduce it to a perfect hierarchy—an arborescence. In an arborescence, all edges point away from the root and there are no horizontal connections, both characteristics we desire in our idealization of what a perfect hierarchy requires. We test our arborescence score in synthetic and real-world directed networks against the current state of the art in hierarchy detection: agony, flow hierarchy and global reaching centrality. These tests highlight that our arborescence score is intuitive and we can visualize it; it is able to better distinguish between networks with and without a hierarchical structure; it agrees the most with the literature about the hierarchy of well-studied complex systems; and it is not just a score, but it provides an overall scheme of the underlying hierarchy of any directed complex network.

## Introduction

Complex networks are a powerful analytical model, used to understand the emergence of complex phenomena arising from the interaction of many different parts in real world systems [1–3]. Hierarchies are one of the many possible properties of real world complex networks. Many complex systems tend to organize themselves in different levels, increasingly centralized [4, 5].

Previous works have identified three different types of hierarchy: order, nested and flow [6, 7]. Order hierarchy is equivalent to node ranking: each node is associated with a score calculated in a given way, and nodes are sorted according to this score (e.g. PageRank [8] and HITS [9]). Nested hierarchy is about finding higher-order structures that fully contain lower order structures, at different levels ultimately ending in nodes. For instance, in hierarchical community discovery one would first group nodes into communities, then group communities in communities of communities, and so on until all nodes of the network are grouped together [10–12]. In a flow hierarchy, nodes in a higher level connect to nodes at the level directly beneath it, and can be seen as managers spreading information or messages to the lower levels.

This works focuses on flow hierarchy in directed networks, because it is the most intuitive concept of hierarchy. A flow hierarchy is how most organizations work, with top level directors passing messages to middle level managers, which then ultimately command teams of people executing the work.

In this paper, we want to estimate the hierarchicalness of a directed network. We start from a directed graph, where each asymmetric edge runs from a higher level node to a lower level one. With this structure as input, we want to compute a score telling us if there is a significant hierarchical structure in the network or not.

Identifying and estimating the hierarchicalness of real world networks has a number of applications. In metabolic networks, hierarchies can help us understand promising pathways for the development of new drugs [13]. Synaptic hierarchies are useful to model how the brain works [14]. Outside the realm of biology, we use social hierarchies to map animal and human dynamics, for instance uncovering patterns in the hiring process of universities [15], or in the purchase patterns of customers in retail [16]. Regardless of the phenomenon represented by the network model—whether it is a social network or a metabolic one—we find hierarchies to be an important piece in understanding the controllability of the system: the agents to influence to make it assume a desired state are usually found in the roots of the hierarchy [17].

We define our score using the concept of arborescence. In graph theory, if node *i* is placed higher than node *j* in the hierarchy we call it *j*’s root. A directed graph that contains exclusively one possible path to go from *i* to *j* is an arborescence [18]. In other words, an arborescence is a directed rooted tree in which all edges point away from the root. Note that, while every arborescence is a directed acyclic graph, not all directed acyclic graphs are arborescences, since they could have multiple roots.

Given any directed graph, we reduce the graph to an equivalent arborescence by collapsing its strongly connected components and identifying the tree’s root. Then, the graph’s hierarchicalness is equivalent to the largest portion of its original edges that can survive the operation. A perfect hierarchy will have a single root and no strongly connected components, thus all edges survive the operation and we obtain an arborescence score of one. A strongly connected component has no hierarchy by definition, since messages originating from any given node can reach any other given node: all edges will be lost, implying an arborescence score of zero.

Our arborescence score is akin to the Levenshtein—or edit—distance [19] between the original graph and its largest possible arborescence. In fact, we can call it a “graph edit distance” [20].

This is not the first paper addressing the problem of estimating the flow hierarchy of directed complex networks. We are aware of three major techniques to address the problem. The first starts by estimating the “agony” of a graph: the number |*E*_*a*_| of edges connecting a lower rank node to a higher rank nodes (i.e. a backward edge) [21]. Ranks are established so that |*E*_*a*_| is minimized. The hierachicalness of *G* is then 1 − (|*E*_*a*_|/|*E*|), where |*E*| is the number of edges. The second calculates the fraction of edges not participating in cycles in a directed graph: the higher this fraction, the more a network is hierarchical [22]. This is known as “flow hierarchy” (FH). The third, “global reaching centrality” (GRC), is quantified by identifying the node which can reach the largest fraction of nodes in the network via outgoing edges [6]. The more distinct this root node is from the rest of the network, the more the structure is hierarchical, because there is one node “commanding” it. In this paper, we compare our arborescence score with these alternatives, showing the strengths and weaknesses of each method in different types of scenarios, using both synthetic and real-world directed networks.

Our results show that the arborescence score is a very demanding test for a network, usually returning lower scores across the board. However, it has three strengths making it an advancement to the state of the art.

First, it is better able to distinguish between random networks with no hierarchy and scale-free networks with a hub-spoke hierarchy. This makes it able to align with the literature in describing the hierarchies of some well-studied complex systems, recognizing hierarchies for textbook cases of hierarchical networks that other methods fail to detect.

Second, the algorithm for the computation of the arborescence score is graphic, meaning that it can be visualized. It consists in identifying strongly connected components and edges against the flow. This enables possible suggestions for operations to strengthen—or to destroy—the hierarchy.

Finally, the output is richer than the alternatives. FH only returns a number, estimating the hierarchicalness of a network. GRC provides a local score for each node, adding an order hierarchy besides its overall estimation of the flow hierarchy of the network. Agony places each node in a level, but how the levels connect to each other is unknown. Our method can return a full arborescence view of the network, placing nodes into layers like agony, but also connecting them to their predecessors and descendants. This view can be used to have a deeper understanding of the hierarchy underlying the phenomenon represented by the original network.

For reproducibility purposes, we release the code of our method and the data from the experiment section as S1 File in the Supporting Information, and online for download (http://www.michelecoscia.com/?page_id=1273).

## Methods

In this section we detail the procedure we design to go from a directed graph to its arborescence score. As stated in the introduction, our hierarchicalness score is simply the fraction of edges that survive when transforming a directed graph into its corresponding arborescence. We start by defining more precisely what an arborescence is. Then, we present the three steps of the algorithm: condensation, rooting and the calculation of the score itself.

### Preliminaries

Assume a directed graph *G* = (*V*, *E*), with *V* being a set of nodes and *E* a set of directed edges. Assume *i*, *j* ∈ *V* being two nodes of *G*. If there is an edge connecting *i* to *j*, then (*i*, *j*) ∈ *E*. Since *G* is directed, if *j* does not link back to *i*, then (*j*, *i*) ∉ *E*. In fact, in a directed graph, (*i*, *j*) ≠ (*j*, *i*).

The terms “arborescence”, “directed tree”, and “directed acyclic graph” are related and sometimes colloquially used interchangeably. Since the distinction between them is important for this paper, we define them formally, and then we provide a quick example to aid the reader in telling them apart. We also define what an “arborescence forest” is, since the concept is useful for the remainder of the paper. We build up from the definition of a strongly connected component.

**Definition 1 (Strongly Connected Component)** *Let P be the set of all possible paths following the edge directions in a directed graph G. A Strongly Connected Component is a subset V′ of nodes in G such that*, ∀*i, j* ∈ *V*′ *both p = (i → … → j) and p′ = (j → … → i) are in P*.

**Definition 2 (Directed Acyclic Graph)** *A Directed Acyclic Graph is a directed graph G that has no strongly connected component*.

**Definition 3 (Directed Tree)** *Let W be the set of all possible simple walks ignoring the edge directions in a directed graph G, where a simple walk is a path in which no edge (i,j) is present more than once. A Directed Tree is a directed acyclic graph G in which ∄w∈W such that the starting node and the ending node are the same, i.e. there are no simple cycles*.

**Definition 4 (Arborescence)** *An Arborescence is a directed tree G in which all nodes have in-degree of one, except the Arborescence root, which has in-degree of zero*.

**Definition 5 (Arborescence Forest)** *An Arborescence Forest is a graph G with multiple weakly connected components, each one of them being an arborescence*.

Arborescences have several properties which derive from their definition. If a node *i* is in a higher level than a node *j*, then the edge (*j*,*i*) cannot be part of *E*. If *i* is the root of the arborescence, then there always is one and only one path going from *i* to any other node in the graph. In an arborescence, all edges have to point away from the root.

**Fig 1:**
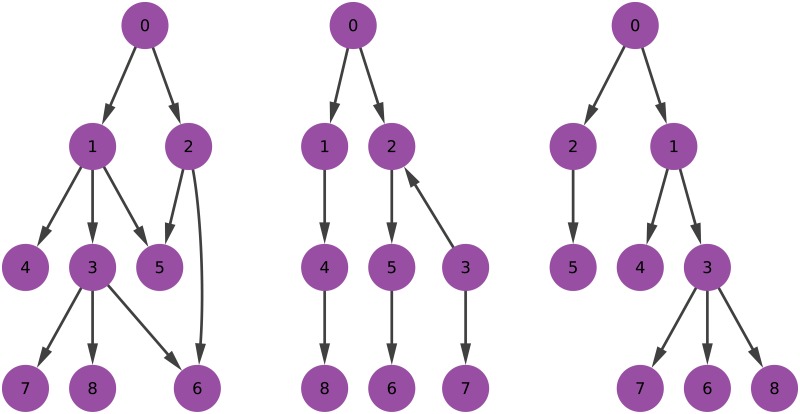
Examples of directed acyclic graphs in increasing levels of structural strictness. From left to right: a directed acyclic graph, a tree, and an arborescence.

Fig 1 depicts the graphical examples of these structures.

On the left we have a direct acyclic graph. If we follow each edge’s direction, we are never able to return to the node that we started at. Since a cycle is defined as a path that allows to eventually reach a node from itself, it is easy to see that the graph on the left has no cycles, hence we call it “acyclic”. The graph is not an arborescence because there are nodes with in-degree higher than one (e.g. 5 and 6). The graph is also not a directed tree: if we were to ignore the direction of the edges, there would be multiple cycles in the graph, one of them being: 0 → 2 → 5 → 1 → 0.

The example in the middle of Fig 1 is a directed tree—and so, by definition, a directed acyclic graph. If we were to ignore the edge directions, there would be no cycle in the graph. However, this is not an arborescence. In the graph, we break the requirement of having a maximum in-degree of one. In fact, we cannot even decide which node is the root of the graph, since both node 0 and node 3 have in-degree of zero.

Finally, the example on the right in Fig 1 is an arborescence—and, by definition, a tree and a directed acyclic graph. There is a single root with zero in-degree (node 0), and the rest of nodes have in-degree of one. As a consequence, there is exactly one path leading from the root to each of its descendants.

If we were to transform all these graphs into arborescences, we would have to delete the offending edges. In the example on the left, we would have to delete two edges: one between (1,5) and (2,5), and one between (2,6) and (3,6). According to the intuition of the arborescence score given in the introduction, the graph will then have a score of.8, since we will end up with an 8-edge arborescence from the 10 edges in the original directed acyclic graph. The middle graph will score .875: we need only to delete the offending (3,2) edge, preserving 7 edges out of 8. Note that we are allowing multiple arborescences components, thus it is more precise to say that we transform the graph into an arborescence forest. There is no need to delete any edge from the rightmost graph, which then has an arborescence score of 1.

Note that, if DAG is the set of all directed acyclic graphs, T is the set of all directed trees, and A is the set of all arborescences, then DAG⊂T⊂A.

### Step #1: Condensation

An arbitrary directed graph can contain multiple strongly connected components. A strongly connected component is a set of nodes such that there is a path in each direction between each pair of nodes in the component. It is easy to see that strongly connected components cannot be part of a perfect hierarchy. A strongly connected component does not have a root, because each node can reach any other node. It does not have any levels either. Therefore, an arborescence cannot contain a strongly connected component. If we want to transform a directed graph into its corresponding arborescence, we need to remove its strongly connected components.

Such removal happens as the first step of our algorithm. We perform it by means of graph condensation. In graph condensation, we firstly detect all the strongly connected components in the graph. Then, each of these components is collapsed into a single node, which represents all the nodes in the component. All edges pointing to a node in the component will point to this “super node”. The super node inherits also the out-going connections of the nodes it contains. Once the graph condensation operation is complete, the result will be a directed acyclic graph.

This operation can be interpreted in the following way. Suppose the graph represents an organization, where the nodes are employees and they point to the people they work with. A strongly connected component can be thought of as a set of people who have working relations with each other. This can be called a “team”, and it can be considered effectively as a horizontal sub-unit in the organization. For all matters and purposes, the team acts as a single unit, and therefore can be considered as a single node in the network. The condensation step makes sure that all “teams” are collapsed in their own nodes in the organization’s organigram.

Note that there are alternative ways to reduce an arbitrary directed graph to a corresponding directed acyclic version [23, 24]. For this paper, we stick to graph condensation since it fits our intuitive explanation of hierarchies. We plan to explore such alternative approaches as future work.

### Step #2: Rooting

We now have to transform the directed acyclic graph into an arborescence forest. We call this process “Rooting”, because it is equivalent to defining the root(s) of the graph. To do this, we want to take the DAG and remove (1) undirected cycles, and (2) edges pointing to the root. To perform both operations at the same time we recall the salient property of all arborescences: the maximum in-degree in the graph must be equal to 1. Therefore, we have to cycle over all nodes with in-degree larger than 1 and delete incoming edges until this property is satisfied.

However, we cannot pick the edges to be deleted at random, because we might end up with edges pointing toward the root instead of away from it. Here, we use closeness centrality. The closeness centrality *C*_*i*_ of node *i* is defined as the reciprocal of the sum of the shortest path distances from node *i* to all other nodes *j* ∈ *V* reachable from *i* [25]. Formally:

$$C_{i}=\frac{1}{\sum_{j\in V}d(i,j)},$$

where *d*(*i*, *j*) is the number of edges to go from *i* to *j*, undefined if there is no such path. If node *i* is a leaf, meaning it has no out edge, all *d*(*i*, *j*) are undefined. In that case, *C*_*i*_ = 0.

For each node with in-degree higher than one, we will keep only the connection coming from the node with the lowest *C*_*i*_. We preserve the lowest centrality connection because the nodes closer to the root will tend to have lower centrality. This property originates from the *C*_*i*_ definition: these nodes will have more possible paths, and these paths will be longer. The paths are longer because, being closer to the root, the node will have more levels beneath itself.

The rooting process is order-independent: the order with which we consider each node for the edge removal does not change the end result. This is because we already know the number of edges that needs to be preserved, which is the number of nodes minus one.

Note that in the rooting process we might obtain multiple roots. This is a desirable property: the rooting process will not purge entire branches of the network only because there is a single misaligned edge. After rooting, we might discover that the graph contained multiple arborescences. The process will result in a graph with multiple weakly connected components, each one of them being a proper arborescence. Thus, the result is an arborescence forest.

### Step #3: Arborescence score

From the previous step, we obtained an arborescence forest, i.e. a graph whose all connected components are arborescences. We are now ready for the last step of the process: the actual computation of the score. We take our inspiration from the Levenshtein edit distance: a measure of the dissimilarities between two strings that counts the minimum number of operations required to transform one string into the other. Here, the arborescence score is inversely proportional to the minimum number of edges that need to be deleted to transform the original graph into its corresponding maximum arborescence forest.

The number of edges of the original graph is |*E*|. Let G* = (V*, E*) be its corresponding arborescence forest. Our arborescence score *A*_*G*_ is then:

$$\begin{array}{c} A_{G}=\frac{|E^{*}|}{\left|E\right|}. \end{array}$$

*A*_*G*_ takes values from zero—the original graph was a single strongly connected component, thus no hierarchy—to one—the graph was already an arborescence, thus no edge was removed.

**Fig 2:**
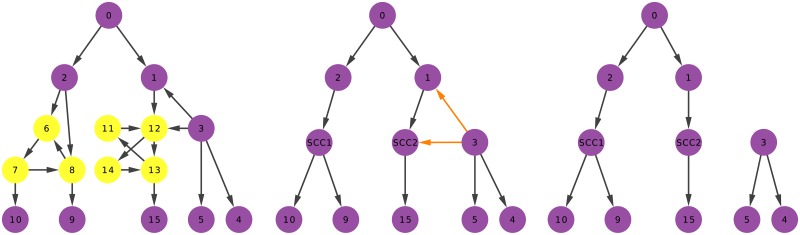
The three steps to calculate *A*_*G*_. From left to right: (left) detection of the strongly connected components (highlighted in yellow); (middle) detection of root-pointing edges for nodes with in-degree higher than 1 (highlighted in orange); (right) the resulting arborescence forest.

Fig 2 depicts a simple workflow of our algorithm. From left to right we apply our three steps. The leftmost graph is the input graph *G*. The graph has 20 edges, thus |*E*| = 20. The graph contains two strongly connected components: (6, 7, 8) and (11, 12, 13, 14). The condensation step collapses them into two single nodes (SCC1 and SCC2), collapsing also the incoming and outgoing connections, where appropriate. Then, the nodes with more than one incoming edge identify the one coming from the lowest *C*_*i*_ origin. Finally, after the graph is purged, only nine edges remain, implying |E*| = 9. Thus, *A*_*G*_ = |E*|/|*E*| = 9/20 = 0.45.

## Results

In this section we provide some results to highlight the strengths and weaknesses of the arborescence score for the detection of hierarchies. We compare mainly with three other state of the art approaches: Agony (AGO) [21], Flow Hierarchy (FH) [6], and Global Reaching Centrality (GRC) [22]. We use Tatti’s optimized implementation of Agony [26](http://research.cs.aalto.fi/dmg/software.shtml). FH is implemented in the current version of the networkx Python package (http://networkx.github.io/), while GRC’s function is written and scheduled for version 2.0. We use these implementations for the comparison. We implemented the arborescence score in a stand alone Python module. The module is available for download, together with the data used for the experiments in this section (http://www.michelecoscia.com/?page_id=1273).

We start with toy examples to show some salient features of the three methods. We then move to experiments with larger synthetic networks, to highlight the resilience of each method to random networks with and without hierarchical structure. We then move to case studies on real world networks. In the rest of the paper we use the terms “Agony” and “AGO” to refer to the hierarchical score obtained using the Agony measure, not the Agony measure itself. If |*E*_*a*_| is the Agony measure, what we call “Agony” from now on is 1 − (|*E*_*a*_|/|*E*|).

### Toy examples

**Fig 3:**
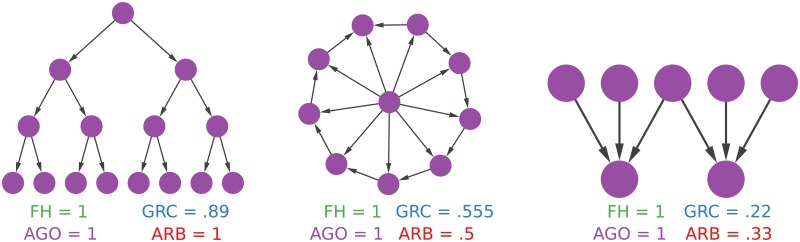
Three toy examples showing different types of directed graphs, with different levels of hierarchy. From left to right: a balanced arborescence with height = 3 and branching factor = 2; a wheel graph, with a hub connecting to all nodes in a circle with a flipped edge; a small directed hierarchy with multiple roots.

Toy examples are a way to infer what each score means: what do the different measures really find? We provide three simple examples. Fig 3 depicts them.

From Fig 3 we can see a few things. One of the main weaknesses of GRC is that it sometimes fails to give a perfect hierarchy score to structures that humans would consider perfect hierarchies. This is a property of the formulation of the GRC measure, which can be equal to one only in the case of a star graph [6]—a graph whose hub is connected to all other nodes and no other edges exist. As a result, most perfect hierarchies will score GRC < 1. This is not a problem for the other methods, since balanced trees are arborescences, and by definition they contain no cycles. In both cases, we obtain a perfect score.

The second example shows a weakness of FH. In a wheel graph with a single flipped edge there are many horizontal connections. There is still a root exerting some sort of hierarchical control, but the lower level nodes still link to each other. However, FH only looks for cycles, which are absent from this topology. As result, FH scores 1, although arguably there are horizontal connections in this structure. Both GRC and the arborescence score somewhat penalize this structure. As FH, Agony fails to apply such penalty.

Finally, we show an example of simple hierarchy with multiple root nodes. Again, there are no cycles in the examples, so both Agony and FH still consider this a perfect hierarchy. GRC is the stricter method in this case, while our arborescence score loosely accepts multiple roots.

Summing up, FH and Agony tolerate any kind of pseudo-hierarchy, as long as they do not contain cycles (every directed acyclic graph is a perfect hierarchy); GRC fails to recognize perfect hierarchies and it is more strict for multi-root structures; our arborescence score can find perfect hierarchies like Agony/FH but not GRC, and it is stricter with horizontal connections than with multi-roots hierarchies, avoiding the downsides of Agony/FH.

### Synthetic graphs

How do these methods fare when no hierarchical structure is present at all? It is difficult to find real world networks in which we are confident there is no hierarchy. However, we can generate graphs that should not have any hierarchical structure. Erdős-Rényi graphs should have no hierarchy by definition, since edges are established uniformly at random among the nodes in the graph [27].

**Fig 4:**
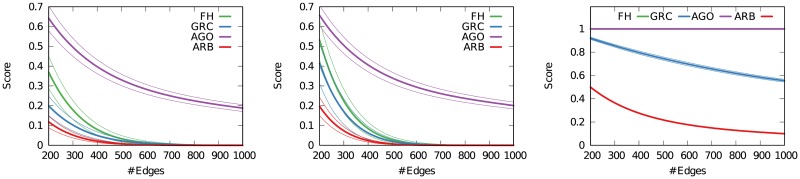
Distribution of hierarchicalness scores according to FH, GRC and arborescence for three classes of random networks for increasing number of edges (number of nodes fixed at 100). From left to right: Erdős-Rényi random graph, Watts-Strogatz small world, and directed scale free network. Average and standard deviation across 100 attempts.

We generate Erdős-Rényi graphs and show in Fig 4 (left) the average score—and score dispersion—of these graphs according to the four measures. We generate 100 graphs with 100 nodes and varying number of edges, since cycles are impossible when there are fewer edges than nodes, and thus a sparse Erdős-Rényi graph could look like a hierarchy. From the figure we can see that all measures, except Agony, correctly give low scores to such graphs, with the arborescence score giving the lowest ones.

We move onto estimating the hierarchy of a Watts-Strogatz small world model [28]. The Watts-Strogatz model is defined as an undirected graph, so we assign a direction randomly to each edge. We set the rewiring probability *p* = .05. The small world model is different from Erdős-Rényi graphs in that it has structural properties: low average path length and high clustering. However, like Erdős-Rényi graphs, it has no hierarchy, as nodes are placed in a low dimensional lattice and connected with a given number *k* of their neighbors, and then a fraction *p* of edges are rewired at random. By performing this test, we verify if the scores can be fooled by the structure imposed by the Watts-Strogatz model. Fig 4 (middle) shows that arborescence is again correct in scoring the lowest among the three options. The scores are slightly higher, but they quickly tend to zero, just like in the Erdős-Rényi case—again with the exception of Agony.

From these two tests, we see that the arborescence score is the strictest method. We have to compare this result with other synthetic networks that exhibit some hierarchical organization. Otherwise, the lower arborescence scores might just be dependent on its strictness, but in relative terms we might not be able to distinguish a random graph from one with a hierarchical structure.

To test how the arborescence strictness affects the scores, we analyze a final class of random graphs: preferential attachment models [29, 30]. We generate a preferential attachment undirected network, and then we establish edge directions always going from the older—and high degree—nodes to the newcomers. This will generate a tree-like structure: in fact, one of the main problems of preferential attachment networks when trying to model real world systems is their lack of cycles and low clustering [31]—both salient characteristics of arborescences.

Fig 4 (right) shows that indeed all the measures score higher for preferential attachment networks. Given the way we generate the preferential attachment networks, we can actually calculate the exact score value for Agony, FH and our arborescence score. By definition, since edges always point outwards from the root, there are no cycles, thus *FH* = 1 and Agony = 1, regardless of the number of edges. For arborescence, if *m* is the parameter of the preferential attachment model indicating how many edges will be attached to a new node, our score will always be ∼1/*m*. It is easy to see why: all the new edges will be pointing to the new node given our wiring rule of imposing the direction from the old node to the new node. So, when rooting the tree, we will have to delete *m* − 1 edges for each node.

We also confirm that the arborescence score is stricter than all the other scores, as suspected. However, this is not a crucial flaw for our method. Suppose that the average score for Erdős-Rényi graphs is *H*_*ER*_ and for preferential attachment graphs with approximately the same number of nodes and edges is *H*_*PA*_. The *H*_*PA*_/*H*_*ER*_ ratio tells us how much each score *H* will be higher for preferential attachment networks over their corresponding no-hierarchy graphs. We want a high ratio value, because we expect *H*_*ER*_ = 0 and *H*_*PA*_ > 0.

**Fig 5:**
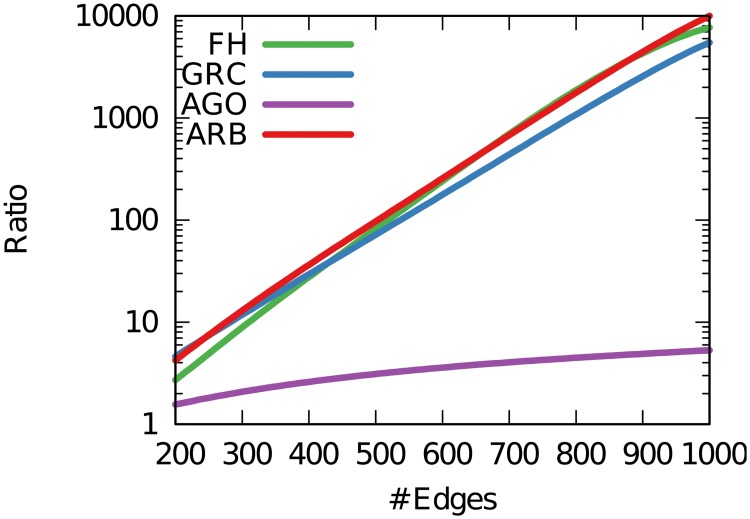
The ratio between each score in an Erdős-Rényi and a preferential attachment graph (*H*_*PA*_/*H*_*ER*_) with 100 nodes, for increasing number of edges.

From Fig 5 we see that FH can hardly distinguish between an Erdős-Rényi and a preferential attachment graph when the average degree is low. GRC and our arborescence score are tied when it comes to this scenario: they are both able to make that distinction. As the number of edges grows, FH and arborescence scores are better able to tell the difference between the no hierarchy and the hierarchy case. As expected, Agony performs particularly poorly given the high scores it returns for Erdős-Rényi graphs.

Summing up, the arborescence score is the strictest method, giving lower scores. However, it is better able to identify networks with a complete lack of hierarchy, and it is better suited to distinguish them from networks with some sort of hierarchy—for instance preferential attachment networks—regardless of their edge density.

### Real world networks

In this section we calculate the hierarchy scores for several real world networks and we compare the results obtained using each one of the methods we discussed so far. Since it is difficult to interpret the score by itself, for each test we compare the result with the expectation given by a null model that is supposed to remove the hierarchy—if present—of a network. We choose relatively small networks because they allow us generate enough null models to estimate the likelihood of observing each hierarchicalness score.

The null model is defined as a double edge switch. We always start from the original network. We randomly select two edges from the network, say *a* → *b* and *c* → *d*, and we exchange the connection. This means that the existing edges are rewired to be *c* → *b* and *a* → *d*. If either of these two edges already existed, the operation is aborted and a new attempt is made. The number of rewiring operations is equal to the original number of edges. There is no rule preventing edges to be rewired back.

The result of this operation is a network in which the in-degree and out-degree distributions are preserved perfectly. Each original node has a corresponding node with the exact same in-degree and out-degree, but whose connections are now random and do not respect any hierarchy that could have been there. This is in the same spirit as the configuration model [2], however the configuration model could not be used because it does not always preserve the exact number of edges in the network.

The first result of our investigation is that, notwithstanding all the differences between the methods highlighted in the previous sections, in practice their outcomes are fairly similar. Table 1 shows all the tests we ran. We list all the networks we test, and the result of each method. Most of the studied networks were gathered via the links provided by the Colorado Index of Complex Networks (https://icon.colorado.edu/). We summarize each result with a label representing the comparison of the observed network’s hierarchy score with the score distribution of 1,000 null models.

**Table 1:** The results of the hierarchy null model test for many real world networks. If the observed score is at least 2 standard deviations higher than the null models’ scores then we say we found a hierarchy (symbol +). If the observed score is 2 standard deviations lower than the null models, we say we found an anti-hierarchy (symbol −). Otherwise there is no hierarchy (symbol ×). * marks networks for which we first calculated the network’s backbone using [41]. If the last column has a ✓, it means that there is at least a three-to-one agreement on the structure of the network and our arborescence method is in the majority. For all networks, we report the number of nodes (*V*), edges (*E*), edges in the reduced graph (E*) and arborescence runtime in seconds (*t*).

Network	*V*	*E*	E*	*t*	FH	GRC	AGO	ARB	Agreement?
Colombia Social [32]	863	9,639	131	0.021	−	×	−	−	✓
Colombia Mobility [32]	863	6,614	22	0.012	×	×	×	×	✓
UN Migration Stocks*[33]	175	1,546	8	0.002	×	×	×	×	✓
O*Net*[34]	496	2,848	14	0.005	×	×	×	×	✓
C. Elegans Frontal [35]	131	764	14	0.002	+	×	+	×	
Hiring Business [15]	113	3,515	40	0.005	+	+	+	+	✓
Hiring CS [15]	206	3,053	77	0.006	+	+	+	+	✓
Hiring History [15]	145	2,496	60	0.004	+	+	+	+	✓
Literature Citation [36]	118	613	613	0.012	+	+	+	+	✓
Literature Criticism [37]	35	81	79	0.002	×	×	×	×	✓
Physician Trust [38]	241	1,098	71	0.004	+	+	×	+	✓
Foodweb Everglades [39]	69	916	26	0.002	+	+	+	+	✓
Foodweb Maspalomas [39]	24	82	37	0.001	+	+	+	+	✓
Foodweb Chesapeake [39]	39	177	60	0.001	×	+	+	+	✓
Foodweb ChesLower [39]	37	178	34	0.001	×	+	+	+	✓
Foodweb ChesMiddle [39]	37	209	21	0.001	×	+	+	+	✓
Foodweb ChesUpper [39]	37	215	26	0.001	+	+	+	+	✓
Foodweb ChrystalC [39]	24	125	7	0.001	×	×	+	×	✓
Foodweb ChrystalD [39]	24	100	12	0.000	×	×	×	×	✓
Foodweb Mondego [39]	46	400	27	0.001	+	+	+	+	✓
Foodweb StMarks [39]	54	356	76	0.002	×	+	+	+	✓
Foodweb Michigan [39]	39	221	17	0.001	+	+	+	+	✓
Foodweb Rhode [39]	19	53	24	0.000	+	×	×	×	✓
Foodweb Florida [39]	128	2,106	82	0.004	+	+	+	+	✓
Foodweb Narragan [39]	35	220	10	0.001	+	+	+	+	✓
Yeast [40]	1,870	2,277	2,203	0.051	×	−	×	+	

The method most commonly in disagreement with the majority is FH, which is remarkable given its similarity to our arborescence score—both heavily penalize cycles. This could be interpreted as a strength of our method: while it does not have the disadvantages of GRC—namely GRC’s inability to give a perfect score to a perfect hierarchy—it still agrees with it most of the time.

We now narrow down and expand on two tests: the protein-protein yeast network, because it is the one with maximum disagreement; and the university hiring networks, just as an example in which we can make the case for the arborescence score even when there is agreement with alternative methods. In both cases we run 24,000 additional null models to have crisper distributions of null result scores to discuss.

**Fig 6:**
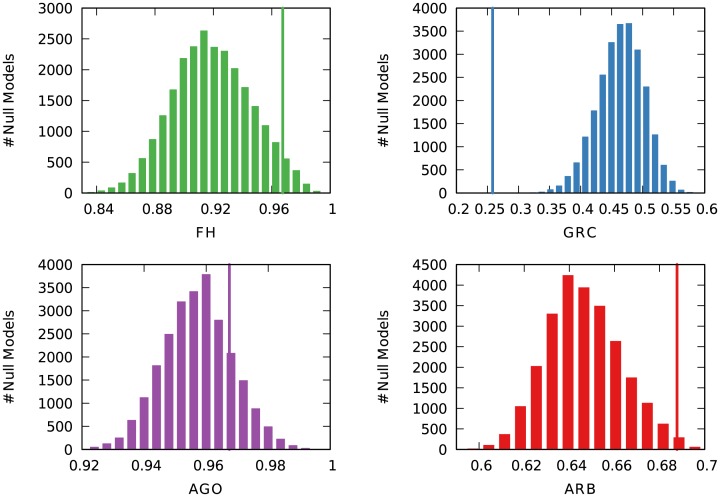
Distributions of null model scores for the protein-protein yeast network. Each hierarchical score, from left to right and top to bottom: flow hierarchy (green), global reaching centrality (blue), agony (purple), and arborescence (red). The y-axis reports the number of null models with a particular hierarchy score (x-axis). The thin vertical line reports the hierarchy score from the observed (non-shuffled) network.

Fig 6 depicts the null model score distribution—and the score for the observed network—for the three methods. We can see that the biggest outlier is GRC: FH and Agony failed to find a hierarchy only because the observed value is less than two standard deviations from the mean, but they still somewhat agree with our arborescence score. By counting the null models outscoring the observation, we can calculate (for FH) a pseudo p-value ∼0.06, just shy of statistical significance. In contrast, the arborescence’s score pseudo p-value is ∼0.017.

In the presence of such disagreement, how can we decide which method provides the most accurate picture of reality? We propose to use the literature’s consensus on this particular network. The network is the protein-protein interaction of *Saccharomyces cerevisiae* [40]. The same network has been used in several studies focused on—or tangentially related to—hierarchies in complex networks. Most studies found it an example of hierarchical organizations of networks [5, 42, 43]. From this exercise we can conclude that our arborescence score outperforms GRC in the single case of disagreement.

**Fig 7:**
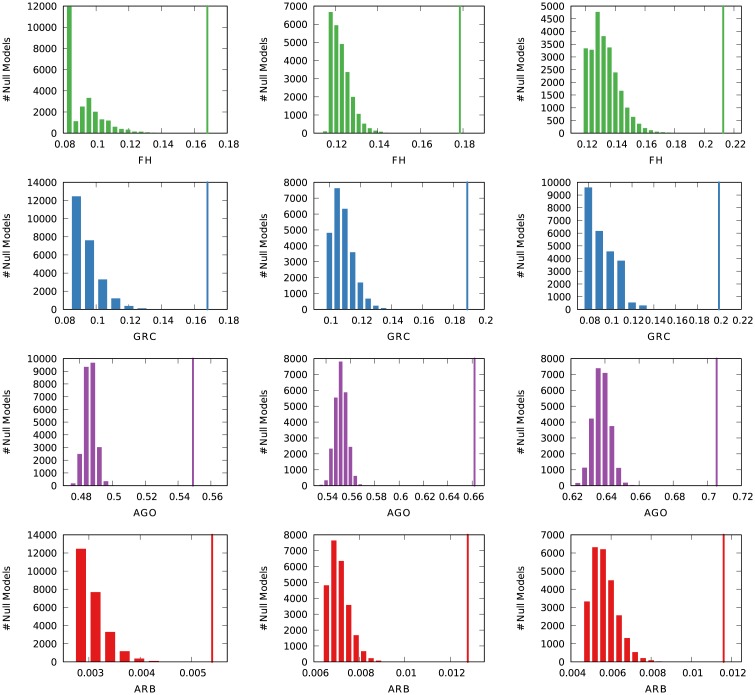
Distributions of null model scores for the three faculty hiring networks, from left to right: Business, computer science, and history. Each hierarchical score appears in a row, from top to bottom: flow hierarchy (green), global reaching centrality (blue), agony (purple), and arborescence (red). The y-axis reports the number of null models with a particular hierarchy score (x-axis). The thin vertical line reports the hierarchy score from the observed (non-shuffled) network.

Fig 7 depicts the null model score distribution—and the score for the observed network—for the university hiring networks [15]. Here, a node is a university and it is connected with a directed edge if a faculty member graduating from it was hired by the other university. In [15], the authors found an unquestionable hierarchical organization of such structures: universities tend to hire almost exclusively from better ranked universities. Unsurprisingly, the measures agree with this statement: all method’s scores in the observed networks are at least five standard deviations away from the null models. However, even in cases of agreement, there is an argument for using different methods for the analysis.

When analyzing a hierarchy, one could be interested in which nodes or connections have the highest impact in the structure. Which one should we delete or rewire in order to generate a non-hierarchical structure? Note that this task is essentially about finding an order hierarchy, and the methods in this paper focus on finding flow hierarchies. One would be better served by using a order hierarchy method, such as the one described in [15]. However, we would expect a flow hierarchy not to be too dissimilar from an order hierarchy, and that is the nature of this test.

Such comparison cannot be performed for FH, because FH just gives an overall score to the entire structure. On the other hand, GRC focuses on identifying the node which can reach the largest fraction of nodes in the network via outgoing edges [6]: operating on such node would have the greatest impact on the hierarchy. Similarly, by reducing the graph to an arborescence forest, our method naturally identifies one or more roots.

Who is at the root in these networks? If we exclude the special “rest of the world” node which lumps together all hiring involving any non-US university, for GRC the nodes with the highest reaching centrality scores are:

- Business hiring network: Washington University, St. Louis (ranked 24th in [15]);
- Computer Science hiring network: University of Maryland, Baltimore County (ranked 133rd in [15]);
- Business hiring network: Southern Illinois University, Carbondale (ranked 133rd in [15]).

None of these nodes is a good candidate for the actual root of the hierarchy, namely the university from which most hiring is drawn. This means that, when it comes to ranking nodes, GRC is very susceptible to noisy connections, the random hiring that defies the hierarchy.

Our arborescence score behaves differently. The noisy connections create cycles, which in turn cause a collapse of the nodes at the top of the hierarchy into strongly connected components in the condensation phase. As a result, the arborescence score returns a (long) list of roots. Differently from GRC—but consistently with Agony, whose output is very similar—these lists always contain the top nodes as ranked by [15]. We can conclude that, even if not specialized for the task of finding an order hierarchy, the flow structure obtained from our arborescence score agrees more than GRC with the order hierarchy found in [15].

### Time complexity

The time complexity of the arborescence score is easy to calculate given that the procedure is divided in three disjoint phases, each of them being well understood. The first phase is graph condensation, which entails finding all strongly connected components in a directed graph. This is done using the Nuutila algorithm, whose complexity is O(V+E) [44]. Step two is rooting, which is done by calculating the closeness centrality of the nodes of the arborescence. To calculate the closeness centrality, one has to find all shortest paths in the network. This is done with the Dijkstra algorithm, whose complexity is $O(E^{*}+V^{*}\log V^{*})$ [45]. Note that this is done on the arborescence, hence the *. In practical terms, E* <<< *E*, thus this second term is dominated by the first. The final step calculating the arborescence score is a simple fraction, thus with complexity O(1). We can conclude that the time complexity of the arborescence score is, for most practical scenarios, O(V+E).

This is the exact same complexity that FH has, since FH aims at finding all cycles in the network and thus can be solved using the very same algorithm of step 1. Its complexity is thus O(V+E). GRC has a higher complexity because it involves the calculation of all shortest paths on the original graph *G*. It also has an extra loop over all the nodes, to find the highest local reaching centrality. Optimizing Agony is know to have $O(E^{2})$ time complexity, since the algorithm relies in finding the maximal Eulerian subgraph of *G*, which has to be recalculated every time we find a backward edge in the remaining part of *G*. Note that we modify Tatti’s implementation in two ways. First, in Tatti’s implementation the input graph is read twice, and we force this to happen once, at the expense of additional memory cost. Second, input and output are forced to be disk I/O operation: we skip both I/O operations by working in memory. We achieve practical speed-ups of up to 33% for large graphs.

**Fig 8:**
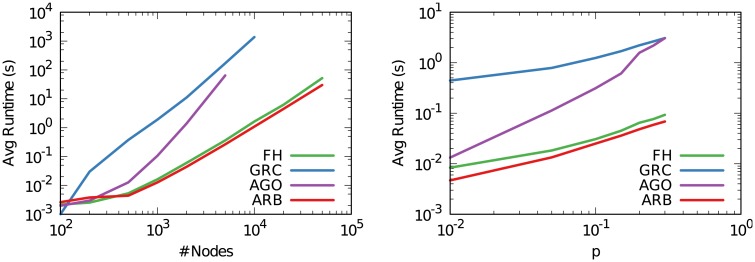
The scalability of the four approaches on synthetic Erdős-Rényi graphs of incresing size. Reported runtimes are the average over 20 independent attempts. (Left) Increasing the number of nodes keeping the connection probability constant (at .01). (Right) Increasing the connection probability, keeping the number of nodes constant (at 500).

Fig 8 depicts the asymptotic time complexity of the three methods on networks of increasing size. Fig 8 (left) shows an asymptote lower than $O(V^{2})$ complexity for the arborescence score and FH, which is expected given that the number of edges goes up quadratically in a random graph whose connection probability is constant (because by increasing *V*, the expected number of edges at constant probability follows O(V(V-1)). Note that FH seems worse than the arborescence score. This is not a significant difference. The reason of this discrepancy is because, in networkx, FH uses the Tarjan algorithm [46] instead of the Nuutila one. The asymptotic complexity is the same, but the multiplicative constant is slightly higher, at least for sparse graphs. GRC shows instead an asymptote $∼O(V^{3})$ and Agony $∼O(E^{2})$, as expected.

Fig 8 (right) confirms that all methods run linearly when changing only the number of edges. The arborescence shows its better multiplicative factor when compared with FH—which tends to catch up for denser graphs –, while GRC has a large overhead.

## Discussion

In this paper we propose a new methodology to estimate the “flow hierarchicalness” of a directed network: how much of the network can be described by top-down flows? There are three main methods adopted in the literature: agony (AGO), flow hierarchy (FH) and global reaching centrality (GRC). We performed three sets of experiments.

In toy examples, we show the intuitiveness of each method, highlighting downsides of each approach. GRC fails to give perfect scores to perfect hierarchies, while FH and AGO do not properly penalize horizontal connections. Our arborescence score is not affected by either issue.

In synthetic networks, we show that all methods but AGO are able to distinguish between random networks without a hierarchy from scale free networks with a hierarchy. This distinction is dependent on the size of a network, with GRC performing better for small networks, and FH for large networks. Our arborescence score is on par with the best alternative on networks of any size.

In real world networks, we show that there is a substantial agreement between methods. This means that no method makes outlandish claims in most practical application scenarios. When there is disagreement, our arborescence score is more in line with the opinion of the established literature on the tested networks. When there is agreement, the arborescence score is more aligned with alternative ways of describing the hierarchy than GRC.

There are several downsides when using our arborescence score, which will require further research. First, our score is very strict. In most practical scenarios, the arborescence of a directed graph will require to delete the vast majority of edges. This is not a problem that can be simply solved by rescaling the scores. The greatest edge losses happen in the collapse phase, which means that the majority of the network is collapsed into a single strongly connected component. This might cause a significant loss of precision when reconstructing the hierarchical structure of the network, which is one of the main contributions of the paper. Alternatives for the collapse phase [23, 24] represent a promising future development of our method.

This issue feeds into the next one: the arborescence representation of a directed graph is a useful structural description of it, but not the most useful. It is currently suitable as a quantitative description—how many edges did we preserve in creating it? –, but when looking at the nodes of the arborescence we can say little about the hierarchy. For instance, in the hiring networks, the arborescence root indeed contains the top order-hierarchy nodes as discovered in [15]. However, the root contains dozens of nodes, and all of them have to be considered peers. There is no objective way to know which of the nodes collapsed in the root is the most important.

## Conclusion

Detecting hierarchies in directed complex networks is a task with many potential applications. It allows us to have a better idea on how natural and man-made phenomena organize themselves. It gives us instruments to enhance our ability to control such complex systems. In this paper, we extend the toolbox available to perform this task. We propose to reduce a directed graph to an arborescence forest—a set of directed trees in which all edges point away from the root—and we propose a score by counting the number of edges preserved by this operation. The more a directed graph is similar to an arborescence, the more hierarchical it is.

There are many possible future developments of this paper. We could tackle the downsides of our arborescence score. We could attempt to relax the requirements of an arborescence, or we could modify our reduction algorithm to preserve more edges from the original network. This line might feed on a second possible extension: reducing a graph to an arborescence is a promising way to have a complete description of the underlying hierarchy of a complex system. We could have a way to detect which nodes are roots, and which nodes are at which level of the hierarchy.

## Supporting information

S1 File Data and code for result replication. The ZIP file contains the data and code necessary to reproduce the figures and tables in the paper, along with an implementation of all the hierarchy methods discussed. The file contains a README file for a deeper explanation on how to use the provided material.
(ZIP)
